# Risk factors for venous thromboembolism in sickle cell disease

**DOI:** 10.1016/j.rpth.2026.103454

**Published:** 2026-03-26

**Authors:** Neha Bhasin, Li Zhang, Ayotola Ajayi, Seva Patel, Taylor Imai, Shreya Agarwal

**Affiliations:** 1 Department of Pediatrics, University of California San Francisco, Oakland, California, USA; 2 Department of Medicine, University of California San Francisco, Oakland, California, USA

**Keywords:** central venous catheter, deep vein thrombosis, pulmonary embolism, sickle cell disease, venous thromboembolism

## Abstract

**Background:**

Individuals with sickle cell disease (SCD) have a high risk for venous thromboembolism (VTE).

**Objectives:**

To determine the associated risk factors and recurrence rate of VTE in SCD.

**Methods:**

We performed a retrospective review of 719 individuals with SCD followed at our institution between 2013 and 2023 and compared those who developed VTE with those who did not.

**Results:**

Of the 719 patients reviewed, 64 (8.9%) patients had 71 VTEs during the study period; 28% of the VTE events were pulmonary embolism and 61% were deep vein thrombosis. The odds ratio (OR) for developing a VTE was 164 (*P* < .001) for patients with a history of VTE, and the VTE recurrence rate was 31% during the study period. In addition, a history of stroke (OR, 39.8; *P* < .001) and a record of 6 or more hospitalizations in a 12-month period (OR, 15.5; *P* < .001) were significant risk factors for VTE in SCD. Other risk factors included smoking (OR, 7.2; *P* < .001), presence of a central venous catheter (OR, 6.9; *P* < .001), and use of hormonal therapy (OR, 9.6; *P* < .001).

**Conclusion:**

We conclude that the risk of VTE in SCD is associated with a history of VTE or stroke, increased health care utilization, use of CVCs, hormonal therapies, and smoking. The high VTE recurrence rate in this study suggests a need to better prevent and treat VTEs in individuals with SCD.

## Essentials

• Individuals with sickle cell disease (SCD) have a high risk for venous thromboembolism (VTE).
• We aimed to determine the associated risk factors and recurrence rate of VTE in SCD.
• Prior VTEs, stroke, and high hospitalization rates increase risk of VTEs in SCD.
• VTE recurrence is extremely high in SCD.

## 1 Introduction

Sickle cell disease (SCD) is an autosomal recessive genetic disease that affects 1 in 500 African American births and 1 in 36,000 Hispanic American births. Approximately 120,000 individuals in the United States and 8 million people worldwide are affected [1,2]. SCD causes chronic hemolysis and recurrent episodes of vaso-occlusion that adversely impact the systemic and pulmonary vasculature. SCD is associated with an increased risk of venous thromboembolism (VTE), a disorder that includes both deep vein thrombosis (DVT) and pulmonary embolism (PE) [3].

VTE is associated with more than one-half million hospitalizations in the United States each year and is a contributing cause in 100,000 more deaths. It causes long-term morbidity including postthrombotic syndrome following DVT and chronic thromboembolic pulmonary hypertension following PE. Medical costs for VTE in the United States have been estimated to total $5 to $10 billion per year [4]. Observational studies show a 25% increased risk of first VTE in SCD, with a mean age of incidence of 30 years, which is similar to patients with other high-risk thrombophilia and sharply lower than the mean age of 65 years in the general population [5]. VTE associated with SCD is driven not only by coagulation and platelet activation but also by hemolysis-associated endothelial dysfunction, increased erythrocyte adhesion, leukocyte activation causing chronic inflammation, nitric oxide depletion causing vasoconstriction, and free hemoglobin-induced oxidative damage [6,7].

Recurrent VTE is associated with mortality and long-term morbidity in addition to a great burden to the health care system. VTE recurrence risk is determined by the presence or absence of transient risk factors present at the time of the initial VTE event. In a systematic review of the general population, VTE recurrence rates at 24 months were reported to be 0.7% per patient-year for a surgical risk factor, 4.2% per patient-year for a nonsurgical transient risk factor, and 7.4% per patient-year for unprovoked VTEs [8]. A recent retrospective cohort study of individuals with SCD at the State of California database showed a 1-year recurrence incidence of 13.2% and a 5-year incidence of 24.1%; these VTE recurrence rates in SCD are significantly higher than those in the general population [9]. A retrospective review of adult patients with SCD showed recurrence rates as high as 49% over a 15-year period [10], raising concerns that VTE recurrence is a significant health care burden for individuals with SCD.

PE in SCD is a leading cause of death in those with acute chest syndrome (ACS) and may contribute to pulmonary symptoms associated with ACS and pulmonary hypertension [6]. Pulmonary artery thrombosis without lower extremity DVT has been detected in up to 17% of cases of ACS, suggesting that thrombi may formed in the pulmonary arteries of patients with SCD, rather than occurring as embolic events from the distal venous circulation, as commonly seen in the non-SCD population [11]. Sudden death accounts for a considerable proportion of mortality in individuals with SCD, and VTE may therefore be an important and unrecognized cause of morbidity and mortality in SCD and needs further investigation [7].

Patients with SCD often require central venous catheters (CVCs) for chronic transfusions and/or medical management due to poor peripheral vein access. Unfortunately, CVCs are known to be highly prothrombotic with a catheter-related thrombosis rate of 24% in patients with SCD [12]. Despite this known risk, the role of pharmacologic thromboprophylaxis for the prevention of VTE in this setting is uncertain due to a paucity of data related to its risks and benefits.

Given these understudied issues related to VTE in SCD, we conducted a retrospective study in a lifetime sickle cell center at the University of California San Francisco (UCSF), Oakland, to determine the associated risk factors and recurrence rate of VTE in SCD.

## 2 Methods

After institutional review board approval, we reviewed medical records of 823 patients with SCD (all genotypes and all ages) in the UCSF Oakland internal patient database. Patients who had at least 1 comprehensive sickle cell visit during 2023-2024 or were deceased during the study period and had a comprehensive visit within 12 months of death, were included in this study to ensure we had complete medical data for all the patients. Moreover, 104 records were excluded due to missing medical records or patients lost to follow-up during the study period. We conducted our analysis of 719 total patients, among which 64 developed a VTE and 655 did not develop a VTE between May 2013 and May 2023.

### 2.1 Data collection

Data collected via review of electronic medical records included age, sex, race, body mass index, and sickle cell genotype. We reviewed the clinical history of all patients including a history of overt or silent stroke, VTE prior to the study period, CVC use during the study period, splenectomy, pregnancy, surgeries, smoking, the number of hospitalizations, and use of hormonal therapies in women. Outpatient visits including infusion center visits for transfusions or pain management were not included as hospitalizations. For patients who developed a VTE during the study period, we collected data related to the type and location of the VTE, anticoagulation therapies used, if available, and complications of bleeding, if any. If there was a recurrent VTE during the study period, defined as a new VTE 6 months after the initial VTE within the study period, we collected information about the timing and location of the recurrent VTE as well.

### 2.2 Statistical analysis

Median with IQRs were used to describe the continuous variables, and counts with percentages to describe the categorical variables. To compare variables between those with and without VTE, Wilcoxon rank sum test for the continuous variables and chi-squared test for the categorical variables were used. Statistical significance was declared based on *P* value <.05. Furthermore, we used univariable and multivariable logistic regression modeling to identify the risk factors associated with VTE for all individuals and univariable logistic regression modeling for female patients separately. Statistical significance was declared based on *P* value of <.05. No multiple testing adjustments were conducted. All analyses were done by R software, version 4.3.2 (R Foundation for Statistical Computing).

## 3 Results

### 3.1 Patient characteristics

Demographics of the study population are shown in Table 1. Of 719 total patients, VTE occurred in 64 (8.9%) patients overall, in 2 of 280 patients (incidence, 0.7%) under the age of 18 years, 15 of 142 patients (incidence, 10.6%) aged 18 to 25 years, and 47 of 297 patients (incidence, 15.8%) aged 26 years and older (Table 1; *P* < .001). There was no difference in sex and race between the VTE and the non-VTE groups. Two patients had a VTE under the age of 18 years; 1 patient had a recurrent VTE following a prior motor vehicle accident-associated VTE, and the other patient under 18 years was a pregnant female patient with a catheter associated VTE.

**Table 1:** Patient demographics in VTE and non-VTE groups.

Characteristic	Patients with VTE (*n* = 64)	Patients without VTE (*n* = 655)
Age (y), median (IQR)	33 (25.5-42)	20 (12-32)
<18	2 (3.1)	278 (42.4)
18-25	15 (23.4)	127 (19.4)
>25	47 (73.4)	250 (38.2)
Sex		
Male	31 (48.4)	315 (48.1)
Female	33 (51.6)	340 (51.9)
Race		
Black or African American	61 (95.3)	535 (81.7)
Hispanic or Latino	1 (1.6)	2 (0.3)
Asian	0	5 (0.8)
White or Caucasian	0	10 (1.5)
Unknown	2 (3.1)	103 (15.7)

Values are given as *n* (%) unless specified.

VTE, venous thromboembolism.

### 3.2 Clinical characteristics

Clinical characteristics of the patients in the 2 groups are shown in Table 2, with the odds ratio (OR) calculated for each patient characteristic in Table 3 and for female patients only in Table 4. Most patients in both groups had the HbSS genotype of SCD. Twenty-two patients (34.4%) in the VTE group and 2 patients (0.3%) in the non-VTE group had a history of VTE prior to the study period (OR, 163.6; 95% CI, 33.34-1288.53; *P* < .001; Table 3). The risk of VTE also was higher in patients with a history of overt stroke (OR, 39.8; 95% CI, 8.6-209.3; *P* < .001; Table 3). The number of hospitalizations 12 months prior to the initial VTE in the VTE group were compared with the number of hospitalizations in a 12-month period (May 2022-May 2023) for the non-VTE group (Table 2). We capped the number of hospitalizations at 6 or more hospitalizations as hospitalizations beyond 6 could not be captured reliably. We found that the OR of having 6 or more hospitalizations in the VTE group compared with that in the non-VTE group was 15.5 (95% CI, 4.49-55.04; *P* < .001; Table 3).

**Table 2:** Clinical characteristics in VTE and non-VTE groups.

Characteristic	Patients with VTE (*n* = 64)	Patients without VTE (*n* = 655)
BMI (kg/m^2^)		
Underweight (<18.5)	6 (9.4)	202 (30.8)
Healthy (18.5 to <25)	27 (42.2)	239 (36.4)
Overweight (25 to <30)	17 (26.6)	107 (16.3)
Obese (≥30)	14 (21.9)	45 (6.8)
Unknown	0	62 (9.5)
Genotype		
Hb SS	48 (75)	396 (60.5)
Hb Sβ^0^	1 (1.6)	16 (2.4)
Hb Sβ^+^	4 (6.2)	53 (8.1)
Hb SC	11 (17.2)	160 (24.4)
Other	0	30 (4.6)
History of VTE	22 (34.4)	2 (0.3)
Pregnancy during study period^a^	3/32 (9.4)	45/340 (13.2)
Hormonal therapy use^b^	13/33 (39.4)	18/340 (5.3)
Surgery	Past 30 d: 6 (9.4)	Any surgery:42 (6.4)
Splenectomy	11 (17.7)	78 (12)
Smoking	13 (20.3)	64 (9.8)
Overt stroke	17 (26.6)	6 (0.9)
Silent stroke	6 (9.8)	23 (3.5)
Hospitalizations in the past year		
0	27 (42.2)	468 (71.5)
1	3 (4.7)	103 (15.7)
2	5 (7.8)	38 (5.8)
3	2 (3.1)	12 (1.8)
4	6 (9.4)	9 (1.4)
5	3 (4.7)	5 (0.8)
≥6	18 (28.1)	17 (2.6)
Central venous catheter present	35 (55.6)	66 (10.1)

Values are given as *n* (%).

BMI, body mass index; VTE, venous thromboembolism.

a There were 48 total pregnancies among the included women.

b Hormonal therapy use was noted in 31 women.

**Table 3:** Odds ratios^a^ of patients’ demographic and clinical characteristics for all patients based on univariable and multivariable logistic regression.

Characteristic	Univariable analysis	*P*	Multivariable analysis	*P*
Sex				
Male	Reference group		Reference group	
Female	0.98 (0.59-1.65)	.948	0.72 (0.30-1.71)	.465
Race				
Black or African American	Reference group		Reference group	
Other	0.22 (0.05-0.60)	.011	0.15 (0.01-0.85)	.060
BMI (kg/m^2^)				
Normal, 18.5 to <25	Reference group		Reference group	
Obese, >30.0	2.75 (1.31-5.59)	.006	2.92 (0.71-10.62)	.114
Overweight, 25 to <30	1.41 (0.72-2.67)	.303	1.17 (0.39-3.30)	.773
Underweight, <18.5	0.26 (0.10-0.61)	.004	0.23 (0.04-0.98)	.075
Genotype				
Hb SS	Reference group		Reference group	
Hb S β thalassemia	0.60 (0.20-1.42)	.291	0.54 (0.07-2.33)	.460
Hb SC	0.57 (0.28-1.09)	.106	0.43 (0.12-1.28)	.149
History of surgery 30 d prior to VTE	1.61 (0.59-3.69)	.302	0.19 (0.04-0.79)	.033
History of splenectomy	1.58 (0.75-3.05)	.197	1.04 (0.28-3.31)	.945
History of smoking	**6.27 (3.54-11.00)**	**<.001**	**7.22 (2.95-18.44)**	**<.001**
History of overt stroke	**38.94 (15.40-112.26)**	**<.001**	**39.79 (8.60-209.33)**	**<.001**
History of silent stroke	2.99 (1.07-7.23)	.022	0.85 (0.10-5.07)	.872
Hospitalizations in the past 12 mo				
0 to 5	Reference group		Reference group	
≥6	**14.66 (7.08-30.61)**	**<.001**	**15.52 (4.49-55.04)**	**<.001**
CVC in place	**11.08 (6.36-19.51)**	**<.001**	**6.97 (2.85-17.48)**	**<.001**
History of VTE	**170.76 (48.17-1088.72)**	**<.001**	**163.57 (33.34-1288.53)**	**<.001**

Values are given as odds ratio (95% CI). The odds ratio >5, is in bold.

a The odds ratio of VTE is defined as the probability of VTE divided by the probability of not having VTE.

**Table 4:** Odds ratios^a^ of patients’ demographic and clinical characteristics for female patients based on univariable logistic regression.

Characteristic	Univariable analysis	*P*
Race		
Black or African American	Reference group	
Other	0.27 (0.04-0.93)	.080
BMI (kg/m^2^)		
Healthy, 18.5 to <25	Reference group	
Obese, >30.0	2.59 (0.88-7.23)	.072
Overweight, 25 to <30	1.94 (0.82-4.66)	.130
Underweight, <18.5	0.20 (0.03-0.75)	.037
Genotype		
Hb SS	Reference group	
Hb S β thalassemia	0.21 (0.01-1.05)	.135
Hb SC	0.62 (0.22-1.46)	.305
Pregnancy within last 3 mo	0.68 (0.16-2.01)	.536
Hormonal therapy use	**11.63 (4.96-27.17)**	**<.001**
History of surgery 30 d prior to VTE	1.69 (0.38-5.32)	.421
Splenectomy	1.67 (0.59-4.05)	.290
Smoking	**8.62 (3.91-18.96)**	**<.001**
Overt stroke	**36.30 (11.24-140.84)**	**<.001**
Silent stroke	4.30 (1.31-12.22)	.009
Hospitalizations in the past 12 mo		
0-5	Reference group	
>6	**10.56 (3.74-29.23)**	**<.001**
CVC in place	**11.13 (5.13-24.72)**	**<.001**
History of VTE	**63.37 (15.31-431.52)**	**<.001**

Values are given as odds ratio (95% CI). The odds ratio >5, is in bold.

BMI, body mass index; CVC, central venous catheter; VTE, venous thromboembolism.

a The odds ratio of VTE is defined as the probability of VTE divided by the probability of not having VTE.

Additional known risk factors for VTE included CVCs (OR, 6.97; 95% CI, 2.85-17.48; *P* < .001; Table 3), a history of smoking (OR, 7.2; 95% CI, 2.95-18.44; *P* < .001; Table 3), and a history of hormonal therapy use (OR, 9.6; 95% CI, 2.17-45.52; *P* < .05; Table 4). Upper extremity DVTs (UE-DVTs) were significantly associated with a CVC (*P* = .001; Table 5). UE-DVT occurred in 27 patients, including 21 who had a CVC and 6 who did not have a CVC in place at the time of the VTE. Of 18 patients who developed PE in isolation, only 7 (20%) had a CVC, compared with 10 without a CVC at the time of the PE event. Even though a history of hormonal therapy was a risk factor for VTEs, we were unable to determine which type of hormonal therapy each patient was taking in the 2 groups. After multivariate analysis, we did not find a strong association between VTEs and silent stroke, obesity, a history of splenectomy, or a history of surgery (Table 3).

**Table 5:** Types of VTE in the CVC vs non-CVC group.

Location of VTE	Total patients (*n* = 64)	Patients with CVC (*n* = 35)	Patients without CVC (*n* = 28)	*P*
UE-DVT	27 (42.2)	21 (60)	6 (21.4)	**.001**
PE	18 (28.1)	7 (20)	10 (35.7)	
LE-DVT	8 (12.5)	0	8 (28.6)	
AV fistula thrombosis	4 (6.2)	1 (2.9)	3 (10.7)	
Intracardiac thrombosis	1 (1.6)	1 (2.9)	0	
Multiple VTEs^a^	6 (9.4)	5 (14.3)	1 (3.6)	

Values are given as *n* (%). The odds ratio >5, is in bold.

AV, arteriovenous; CVC, central venous catheter; DVT, deep vein thrombosis; LE, lower extremity; PE, pulmonary embolism; UE, upper extremity; VTE, venous thromboembolism.

a Six patients had >1 VTE at the same time including 5 patients with 2 VTEs and 1 patient with 3 VTEs simultaneously.

Hydroxyurea use and adherence could not be assessed in our cohort due to significant missing data, but we noted an average HbF level of 7.1% in the VTE cohort within 4 weeks of the VTE event. These data were not available in the non-VTE group. Four patients died during the study period, 1 from multiorgan failure (end-stage renal disease, pulmonary hypertension, and sepsis), 1 from cardiogenic shock, 1 postoperatively after severe ACS and multiorgan failure, and 1 from an unknown cause.

Seventy-one VTE events occurred in 64 individuals during the study period and were confirmed with an imaging modality including ultrasound, Doppler ultrasound, computed tomography scan, echocardiography, or magnetic resonance imaging (Figure). Five patients had 2 VTEs simultaneously, and 1 patient had 3 VTEs simultaneously. We noted that 43.7% (31/71) of the VTEs were UE-DVTs and 28.2% (20/71) were PEs; 78% (21/27) of the isolated UE-DVTs were associated with a CVC compared with 22.2% (6/27) that were not associated with CVC (Table 5; *P* = .001). Only 2 PEs were noted in conjunction with a DVT. Most patients were diagnosed with a VTE when they presented to the emergency department (31; 48%) or were seen in the outpatient setting (6; 9.4%); only 21 (33%) were diagnosed with a VTE during a hospitalization. We could not determine where 6 patients were located during their VTE diagnosis.

**Figure:**
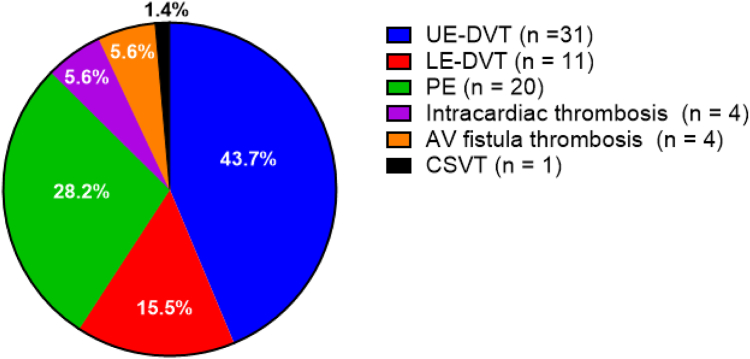
Distribution of VTE subtypes. AV, arteriovenous; CSVT, cerebral sinus venous thrombosis; DVT, deep vein thrombosis; LE, lower extremity; PE, pulmonary embolism; UE, upper extremity; VTE, venous thromboembolism.

After the initial VTE, the follow-up period for each patient was variable. The median follow-up for the patients in the VTE group was 3.3 years (range, 0.2-10.2 years). Twenty-two patients in the VTE group had a prior VTE event prior to the study period, but only 9 patients (41%) were on anticoagulation therapy at the time of VTE recurrence. Twenty patients (20/64, 31%) developed a recurrent VTE within the study period. All patients who developed a VTE were started on some type of anticoagulation therapy, but the exact dates, types, and times of these therapies, including adherence to these therapies, could not be reliably extracted from the medical records. Six patients (9.4%) on anticoagulation therapy developed a bleeding complication. One patient with acute stroke developed a subdural hemorrhage after tissue plasminogen activator was delivered. The other 5 patients had a minor bleeding episodes as defined by the International Society on Thrombosis and Haemostasis (ISTH) [13].

## 4 Discussion

In this retrospective study of 719 individuals with SCD aged 0 to 66 years, 64 patients (8.9%) overall and 14.1% aged 18 years and older developed VTE during the 10-year study period. A history of prior VTE, overt stroke, and high hospitalization rates were significant risk factors for VTEs in SCD in addition to CVCs, smoking, and use of hormonal therapy.

The cumulative incidence of VTE among patients with SCD of 11.2% has been reported in a population-based administrative database from the state of California spanning 23 years [14]. The median age of patients with incident index VTE in the administrative database study was 31 years, which is very similar to the median age of 33 years in our study.

We found that UE-VTEs were strongly associated with CVCs. Prior studies have shown that upper extremity VTEs in SCD are often associated with the presence of indwelling catheters, with recommendations to weigh the risks-to-benefit ratio of indwelling catheter placement in SCD [7,[14], [15], [16]]. However, no well-designed prospective studies have been done to assess the timing of VTEs after CVC placement in SCD or the effectiveness of measures taken to minimize risk of VTEs in patients with SCD and CVCs [17]. This lack of data makes clinical decisions about CVC placement for individuals with SCD very challenging. In the future, the risks of VTE in individuals with SCD should be studied prospectively to determine when the risk of VTE is highest after CVC placement and whether thromboprophylaxis can minimize this risk.

We noted a high number of PEs in our cohort, comprising 28.2% of VTEs, which is similar to a recent retrospective analysis of 48 hospitals in the United States, which observed that 23.8% of VTEs in children with SCD between the years 2009 and 2015 were PEs [18]. In addition, PE without concurrent DVT was common in our cohort; only 2 patients had concurrent DVT and PE. This may be an underrepresentation of concurrent PE and DVT because venous duplex ultrasound was not routinely performed in all patients with PE. Alternatively, the high incidence of isolated PE observed in our study may support the hypothesis that patients with SCD are at risk for thrombi formed in the pulmonary arteries rather than embolic events from the distal venous circulation [6]. A study of the United States National Hospital Discharge Survey reported that the overall incidence of PE was ∼4 times higher in patients with SCD than that in African Americans without SCD [9]. Given the high incidence and incidence, unique pathophysiology, and considerable mortality of PE in SCD (particularly in the setting of ACS), more investigation is needed to determine the proportion of clinically diagnosed PE that represent *in situ* pulmonary artery thrombosis vs embolism from DVT of the extremities.

We observed a high VTE recurrence rate of 31% during the study period. In addition, we noted that even though 22 (34.4%) patients in the VTE group had a history of prior VTE, only 9 patients were on thromboprophylaxis when they were diagnosed with a recurrent VTE during the study period. Because our analysis was retrospective, we did not have reliable data about the management of VTE prior to or during the study period or the use of thromboprophylaxis or adherence to anticoagulation therapies. Our results highlight clear gaps that need to be addressed in future studies to ensure VTE management and prophylaxis strategies are optimized for individuals with SCD. Our current thromboprophylaxis practice includes pharmacologic thromboprophylaxis for all individuals with SCD aged 18 years and older during hospitalizations and indefinite thromboprophylaxis after recurrent VTE for all patients. However, no standard practice exists to ensure adherence to these therapies in the outpatient setting and discussions about the risks and benefits of these therapies. Given our study showed that more than half of the VTEs in individuals with SCD were diagnosed in the emergency room (48%) or in the outpatient setting (9.4%) and only 33% of the VTEs were diagnosed in the inpatient setting, strategies to minimize VTEs in the outpatient setting are necessary going forward as well is optimization of inpatient thromboprophylaxis [9].

Chronic inflammation, endothelial damage, and underlying vasculopathy in SCD in addition to long-term CVC use may play a role in chronic VTE risk in patients with SCD compared with that in the general population where these issues are temporary and mostly occur in the inpatient hospital settings. SCD-associated thrombophilia, therefore, needs to be better studied prospectively to guide thromboprophylaxis in the inpatient and outpatient settings. The use of thromboprophylaxis long-term in individuals with SCD needs to be weighed against its risks with optimal patient education and engagement to ensure optimization of these therapies so recurrent VTEs can be minimized and so can bleeding risks associated with these therapies. In addition, newer anticoagulation agents that minimize risks of bleeding need to be studied in SCD.

Limitations of our study include its retrospective design, variable follow-up period, lack of data regarding anticoagulation therapies. In addition, we lacked ACS events that could be assessed in relation to the PEs noted in our study to assess whether PE in our population was associated with a concurrent ACS event. We also cannot determine the time of first VTE from our study given 34% of our patients with VTE had a history of VTE prior to the study period. All these limitations can potentially be addressed in a prospective study of patients with SCD in the future.

In conclusion, our findings confirm that SCD is a thrombophilia state with a high incidence of VTE, especially in patients with increased hospitalizations, a history of stroke or VTE, use of CVCs, hormonal therapy, and smoking. The high VTE recurrence rate in individuals with SCD and significant VTEs in the outpatient setting suggests a need to better prevent and treat VTE to decrease health care burden in SCD. VTEs are an underrecognized issue in SCD and needs significant attention in the future.
